# Potential role of plasma miR-21 and miR-92a in distinguishing between irritable bowel syndrome, ulcerative colitis, and colorectal cancer 

**Published:** 2020

**Authors:** Elham Ahmed Hassan, Abeer Sharaf El-Din Abd El-Rehim, Emad Farah Mohammed Kholef, Wael Abd-Elgwad Elsewify

**Affiliations:** 1 *Department of Gastroenterology and Tropical Medicine, Faculty of Medicine, Assiut University, Assiut, Egypt*; 2 *Department of Clinical pathology, Faculty of Medicine, Aswan University, Aswan, Egypt*; 3 *Department of Internal Medicine, Faculty of Medicine, Aswan University, Aswan, Egypt *

**Keywords:** miR-21, miR-92a, Colorectal cancer, Irritable bowel syndrome, Ulcerative colitis

## Abstract

**Aim:**

This study aimed to investigate whether plasma miR-21 and miR-92a levels may be used to differentiate between patients with irritable bowel syndrome (IBS), ulcerative colitis (UC), and colorectal cancer (CRC).

**Background:**

miRNA expression profiles are well characterized in CRC, but these expression profiles in UC and IBS remain promising. Screening of high-risk individuals for these diseases has substantial clinical benefits.

**Methods:**

This was a case-control study. We quantified plasma miR-21 and miR-92a expression levels in 100 samples (37 with active UC, 33 with CRC, and 30 with IBS as well as 30 healthy controls) using real-time PCR. Their diagnostic performance for discriminating these diseases was assessed using receiver-operation characteristic curve (AUC-ROC).

**Results:**

The studied miRNAs were differentially expressed among all participated groups. Plasma miR-21 and miR-92a levels exhibited significant upregulation in CRC as compared to IBS, UC, and healthy subjects. Both miRNAs were upregulated in the UC group as compared to IBS and healthy subjects. ROC analysis revealed promising diagnostic performance for miR-21 and miR-92a in discriminating UC from non-UC groups (IBS and healthy subjects) with AUCs of 0.844 and 0.979 respectively. It also distinguished between CRC and UC with AUCs of 0.968 and 0.887 respectively and with reasonable sensitivities and specificities.

**Conclusion:**

Circulating miR-21 and miR-92a can be exploited not only as potential noninvasive biomarkers for detection of CRC, but also for differentiation between functional and organic colorectal disorders.

## Introduction

 Ulcerative colitis (UC) is chronic and recurrent intestinal inflammation which may be associated with serious complications over the long run including colitis-associated colorectal cancer (CACC) (1). The pathogenesis of UC is multifactorial and is not completely understood with genetic, epigenetic, infectious, physiological, and immunological factors being possibly involved (2, 3). Furthermore, the diagnosis, evaluation of severity, and prognosis of UC have remained challenges for clinicians. Lately, there has been a soar of up to 30 times in the incidence of UC and expectedly the CACC incidence (1). The risk of CACC following UC diagnosis is 0.5-1% per year and it rises over time after UC onset (1.6%, 8.3%, and 18.4% over10, 20 and 30 years respectively) (4). CACC estimates 1-2% of colorectal cancers (CRC) that is usually diagnosed at advanced stages (5, 6). It contributes to 15% of mortality in inflammatory bowel disease (IBD) patients with a risk of 1.5–2.4 folds higher than in normal population (5,7).

Recently, a slight decrease has been reported in CACC incidence among IBD patients which may be due to the conventional or biological widespread use therapies and early coloproctectomy, alongside the current guideline recommendation of regular endoscopic screening for early detection (8, 9). Early CACC detection is essential as it carries a high mortality and worse prognosis than sporadic CRC (10). So, newer techniques and approaches e.g., molecular biomarkers have emerged in different biosamples (1). MicroRNAs (miRNAs) are a class of small, non-coding RNAs (approximately 22 nucleotides long) which function as posttranscriptional gene regulators (11). They are involved in the regulation of several biological processes as the cell cycle differentiation, proliferation, immune function, fibrosis, and apoptosis (12). Additionally, they may have an important role in the induction of chronic inflammatory, autoimmune diseases as well as cancer development (13, 14). Furthermore, established functional interactions between miRNAs and pathogenic mechanisms in IBD have been reported by the genome-wide association studies (GWAS) (15). The majority of reports in IBD including UC have been conducted in tissue and cellular cultures, and there are currently few studies on the quantitative assessment of circulating miRNAs in these patients (16, 17). Several articles have reviewed the potential role of some miRNAs in the development of CRC and their relationship with CRC pathogenesis, treatment, and prognosis (14, 18).

Previous studies, focusing particularly on cancer, have demonstrated that miRNAs remain stable in the extracellular space for at least one month, and their circulating profiles can be correlated with tissue miRNA profiles, suggesting the possibility of their use as biomarkers for cancer, tissue/organ damages, or viral infections (18, 19).

As intestinal symptoms are a frequent cause of referrals to gastroenterologists, it is crucial to differentiate between irritable bowel syndrome (IBS) and IBD (20). Previous studies have documented the upregulation of miR-21 and miR-92a in intestinal tissue and cellular cultures of IBD patients (21, 22). Here we tried to examine the expression profiles of miRNAs in the plasma of these organic lesions and to differentiate them from those with functional disorders and their potential explanations. Accordingly, we aimed to assess the expression levels of miR-21 and miR-92a in the plasma of UC and CRC patients compared to IBS and healthy subjects and to evaluate their diagnostic performance as potential non-invasive biomarkers for UC and CRC. 

## Methods

**Study design**

This case-control study was carried out at Assiut University Hospital, Assiut, Egypt, from March 2017 to March 2019. The study was approved by the Local Ethics Committee of Assiut University Hospital and was conducted in accordance with the provisions of the Declaration of Helsinki. An informed consent was obtained from all the participants before enrolment.

**Study population**

This study comprised 100 subjects including 37 treatment-naive patients with active UC, 33 patients with histologically confirmed CRC, and 30 patients with IBS. Patients met the diagnostic criteria for UC based on clinical, endoscopic, and histopathological findings (23) and they had no history of CRC. Criteria of active UC was assessed based on Mayo clinical score (24). Additionally, the diagnosis of IBS was based on Rome IV criteria (25), normal imaging, as well as colonoscopic and histopathological findings. In patients with CRC, the tumor was staged according to the American Joint Committee on Cancer/International Union Against Cancer– the tumor-node-metastasis (AJCC/UICC-TNM) staging system (26). Those patients were selected from the outpatient clinics and the inpatient wards of Gastroenterology and Tropical Medicine Department, Assiut University Hospital, Egypt between March 2017 and March 2019.

Thirty healthy volunteers who were sex and age matched, had no gastrointestinal symptoms and had no prior diagnosis of any other malignancy served as normal controls. Patients with evidence of Crohn's disease, other causes of colitis such as infectious ischemic, or diverticular colitis, familial polyposis, extracolonic malignancy, receiving treatment for CRC as surgical resection, chemotherapy or radiotherapy and pregnant females were excluded.

At study entry, a thorough medical history was taken and physical examination was done for data collection. For example, age, gender and family history of colorectal diseases including IBD, polyposis, and CRC. Blood samples were collected for miRNA analysis. 

**Collection and processing of blood specimens**

Under aseptic precautions, 3 milliliters of venous blood were collected in EDTA tube from each participant to be carefully centrifuged at 4000 rpm for 20 min. Separated plasma were stored at −80°C until the time of analysis.

**RNA extraction and reverse transcription**

Total RNA, containing miRNA, was extracted from plasma samples using the Qiagen miRNeasy Mini Kit (Qiagen GmbH, Valencia, California, USA, catalog no. 217004). The RNA purity was confirmed by the relative absorbance at 260/280 nm. Then, the extracted RNA was stored at -80°C and prepared for usage. Ten µl of the extracted RNA was used for cDNA synthesis using miScript II reverse transcription (RT) Kits (Qiagen GmbH, Valencia, California, USA), under the reactive condition of 37°C for 60 min, then 95°C for 5 min. Then, miScript SYBER Green PCR kits were used with miScript Primer Assays according to the real-time PCR Amplifier manufacturer's instructions (Qiagen GmbH, Valencia, California, USA, catalog no. 218073). The mature miRNA-21 and miRNA-92a sequences were (UAGCUUAUCAGACUGAUGUUGA) and (AGGUUGGGAUCGGUUGCAAUGCU) respectively with Housekeeping gene as an endogenous control. The reactivity conditions: initial activation step at 95°C for 15 min, followed by 3-step cycling: 1-Denaturation 94°C for15s, 2-Annealing 55°C for 30s, 3- Extension 70°C for 30s. Cycle numbers: 40 cycles. After reaction, the threshold cycle of fluorescence (Ct) was calculated to further analyze the expression level of miRNA in specimens utilizing endogenous control. A housekeeping gene was added to each sample as an internal negative control (Catalog no. 00033712). The relative expression was expressed by 2-ΔCT (ΔCT= CT target gene - CT reference gene). The relative quantification of each of miR-21 and miR-92a was performed using quantitative real-time (qRT)-PCR according to the manufacturer's instructions. The ∆Ct value was used to represent the relative level of expression of a single miRNA. 

**Statistical analysis**

**Table 1:** Distribution of plasma miR-21 and miR-92a levels across the study population

The study population	Cases (N)	Plasma miR-21 levels (Median & range; Fold changes)	Plasma miR-92a levels (Median & range; Fold changes)
Healthy controls	30	1.22 (0.39 -1.92)	1.23 (0.04 - 1.66)
IBS	30	1.24 (0.39 - 1.94)	1.22 (0.06 - 1.63)
UC	37	1.85 (0.85 - 5.10)	2.48 (1.57 - 12.91)
CRC	33	38.59 (1.67 - 103552.29)	195.3610 (0.21 - 30362.43)

CRC: colorectal cancer; IBS: irritable bowel syndrome; UC: ulcerative colitis

All statistical analyses were carried out using SPSS for Windows version 16 (SPSS Inc., Chicago, IL, USA) and the MedCalc program. The Shapiro-Wilk Test of normality was used to test the normality of data. Quantitative data were expressed as mean ± standard deviation or median and the range (minimum - maximum) for normally or abnormally distributed data respectively, while qualitative data were expressed as percentage. They were compared using Mann-Whitney U test or the Kruskal Wallis test for two or more groups of abnormally distributed data respectively. The receiver operating characteristic (ROC) curves were plotted to measure the diagnostic performance of miRNA expression levels in discriminating patients with UC or CRC and to choose the best cut-off values at which the sensitivity, specificity, positive (PPV) and negative (NPV) predictive value, as well as positive and negative likelihood ratio (+LR, -LR) could be calculated. All tests were two-tailed and statistical significance was assessed at < 0.05.

**Table 2:** Diagnostic accuracy of plasma miR-21 and miR-92a to predict clinical colorectal disease with the best predictive cutoffs

	AUC (95%CI)	SE	SP	PPV	NPV	+LR	-LR
For plasma miR-21							
UC vs. non-UC* (> 1.52 fold changes)	0.844 (0.613 - 0.963)	87.5	91.7	92.9	85.6	10.5	0.1
CRC vs. UC (> 5.1 fold changes)	0.968 (0.854 - 0.996)	93.5	100	100	94.5	-	0.1
For plasma miR-92a							
UC vs. non-UC* (> 1.66 fold changes)	0.979 (0.795 - 0.980)	87.5	100	100	86.7	-	0.1
CRC vs. UC (> 12.91 fold changes)	0.887 (0.745 - 0.965)	83.9	100	100	83.9	-	0.2

* non-UC = irritable bowel syndrome and healthy subjects; AUC: area under the curve; CI: confidence interval; CRC: colorectal cancer; LR: likelihood ratio; NPV: negative predictive value; PPV: positive predictive value, SE: sensitivity; SP: specificity; UC: ulcerative colitis

**Figure 1:**
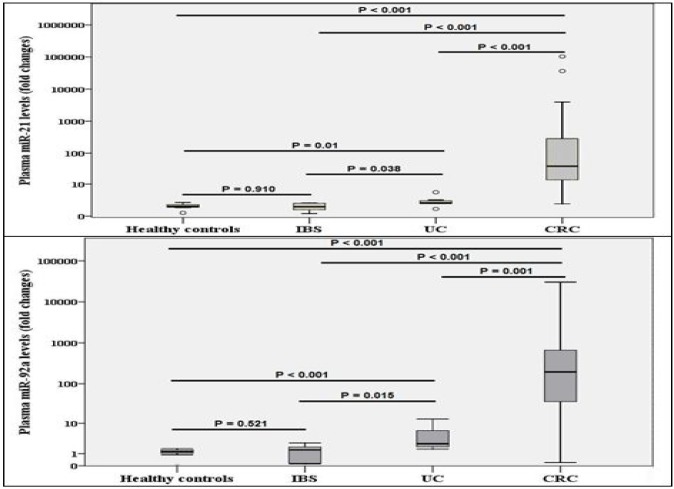
Comparison between different study groups regarding a) plasma miR-21 and b) plasma miR-92a CRC: colorectal cancer; IBS: irritable bowel syndrome; UC: ulcerative colitis

## Results

**Characteristics of the study population**

The present study was performed on 100 patients (51 males and 49 females with mean age of 44.3 ± 14.8 years); 37 patients with UC (15 males and 22 females with mean age of 38.3 ± 17.3 years), 33 with CRC (19 males and 14 females with mean age of 45.3 ± 13.6 years whereas 10, 4,12 and 7 cases were AJCC/UICC-TNM stages I, II, III and IV respectively) and 30 individuals with IBS (12 males and 18 females with mean age of 37 ± 13.5 years) and 30 healthy subjects (17 males and 13 females with a mean age of 41.5 ± 14.4 years) to evaluate the diagnostic value of miR-21 and miR-92a. 

**Plasma expression levels of miR-21 and miR-92a**

In this study, the expression of these 2 miRNAs; miR-21 and miR-92a was detectable in all analyzed plasma samples, as reported in Table 1. There were no significant differences in plasma miRNA levels with respect to age and gender between UC, CRC, IBS patients, and healthy controls (P > 0.05). The expression of the two miRNAs, miR-21 and miR-92a, was significantly elevated in CRC and UC samples when compared with IBS and control subjects (all P < 0.05, Figure 1).

**Diagnostic performance of miR-21 and miR-92a as potential biomarkers for UC and CRC**

**Figure 2:**
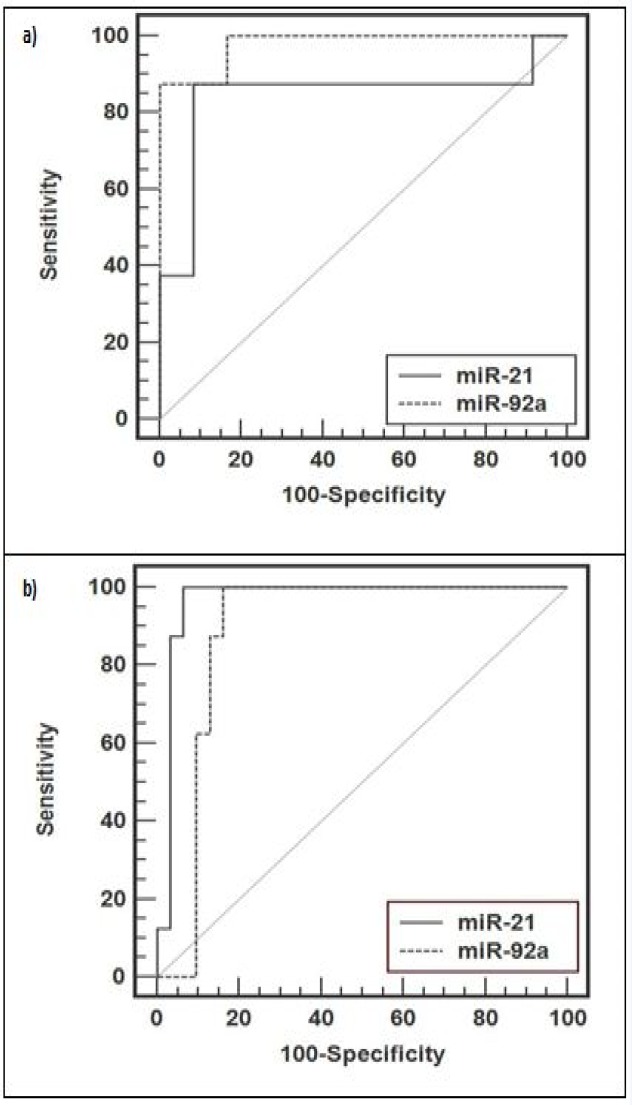
Receiver operating characteristic (ROC) curve analysis using plasma miR-21 and miR-92a for discriminating a) UC from non-UC (IBS and healthy controls) b) CRC from UC

CRC: colorectal cancer; IBS: irritable bowel syndrome; UC: ulcerative colitis

As miR-21 and miR-92a levels were significantly elevated in plasma of UC patients in comparison to IBS and healthy controls, ROC curve analysis was used to explore these circulating miRNAs as potential biomarkers for UC. These analyses revealed that plasma levels of both miR-21 and miR-92a were potential biomarkers for discriminating UC patients from non-UC (IBS and healthy subjects) with an area under the ROC curve (AUC) of 0.844 [95% confidence interval (CI) = 0.713 - 0.963] and 0.979 (95% CI = 0.795 - 0.980), respectively (Table 2 and Figure 2a). At a cut-off value of 1.52 for miR-21, the sensitivity was 87.5% and the specificity was 91.7%. On the other hand, at the cut-off value of 1.66 for miR-92, the sensitivity was 87.5% and the specificity was 100% (Table 2 and Figure 2a). 

Since the expression of miRNAs (miR-21 and miR-92a) in the plasma of patients with CRC was higher than in UC and IBS and healthy subjects, we further assessed the diagnostic value of plasma miR-21 and miR-92a expression levels in the CRC detection. ROC curve analyses revealed that both miRNAs might be helpful to discriminate CRC from UC, with an AUC of 0.968 for miR-21 (95% CI = 0.854 - 0.996) and 0.887 for miR-92a (95% CI = 0.745 - 0.965) (Table 2 and Figure 2b).

## Discussion

In this preliminary study, we carried out a throughput screening of plasma miR-21 and miR-92a expression levels via qRT-PCR analysis in UC, alongside CRC, IBS, and healthy controls. Circulating levels of miRNAs increased in these diseases compared to IBS and controls which may provide insight into the potential biological function of these miRNAs in the pathogenesis of these diseases.

In this study, we found that the expression levels of miR-21 and miR-92a was significantly higher in active UC when compared to IBS and healthy subjects. In addition, both miRNAs were helpful in differentiating UC from non-UC subjects (IBS and healthy controls), where AUC was 0.844 for miR-21 and 0.979 for miR-92a with sensitivity of 87.5% for both and specificity of 91.7 and 100% at cut-off values of 1.52 and 1.66, respectively. These findings were consistent with previous studies reporting increased expression of miR-21 and miR-92a in peripheral blood of UC patients relative to normal controls (17, 22). Wu and colleagues reported that 8 miRNAs including miR-21 were significantly elevated in active UC versus IBS and healthy controls (27). Unlike our results, Ng et al. found that plasma miR-92a over-expression was not associated with UC (18).

The upregulation of these miRNAs in active UC proposes their essential role in UC pathogenesis (21, 22). They can upregulate IFN-γ production and suppress regulatory T cell (Treg) differentiation and hence influence Th1 differentiation and function (27). Dysregulation of miR-21can also promote Th2 cell differentiation, increase intestinal permeability, and promote irreversible fibrosis of IBD predicting its role as a biomarker for disease activity (28). Altered miRNA expression in UC has been associated with other autoimmune-mediated diseases suggesting a generalized inflammatory status (29). Further, its high expression is maintained throughout the development of IBD-associated dysplasia and subsequent carcinogenesis in long-standing disease (30). Studying the role of miRNAs in IBD pathogenesis has sparked an emerging interest in their potential role for microRNA-targeted therapies.

The underlying pathophysiological mechanisms of IBS are still unclear, with some patients with diarrhea-predominant IBS showing serotonergic system dysfunctions associated with increased intestinal permeability or low-grade inflammation (31, 32). The pathophysiologic role of the expression of specific miRNAs in patients with IBS has been reported. Indeed, previous studies revealed that altered miR-510 and miR-29a expression in IBS patients was associated with altered serotonin signaling, intestinal permeability, or even visceral/somatic hypersensitivity (33, 34). To the best of our knowledge, no previous reports investigated the miR-21 and miR-92aexpression in the plasma of IBS patients. Circulating levels of these miRNAs were detectable in IBS samples but were significantly lower in IBD and CRC samples, indicating their potential in differentiating between functional and organic bowel disorders. 

As compared with UC cases, we found that plasma miR-21 and miR-92a expression levels were significantly higher in CRC cases and yielded higher AUCs (0.968 and 0.887) with 93.5 and 83.9% sensitivity; and 100% specificity for both at cut-off values of 5.1 and 12.9, respectively. Similarly, Ahmed et al. found an increased expression of seven miRNAs including miR-21 and miR-92 in the stool of CRC patients compared with that of UC patients (35). 

Several studies have reported that miR-21 and miR-92a levels increased with disease progression from normal to IBD to IBD-associated neoplasia (IBDN), suggesting their involvement in both chronic inflammation and carcinogenesis with their potential value for early colonic cancer detection in this precancerous lesion (30, 35). Also, Ludwig et al. documented that PDCD4/miR-21 dysregulation (increased miR-21and decreased PDCD4 expression) was involved in IBD-associated carcinogenesis (30). Thus, microRNAs as reliable molecular markers should be studied to identify IBD’-associated dysplasia and subsequent carcinogenesis especially for whom surgical management of IBD patients e.g., total colectomy is mandatory. Further, early detection of colitis-associated colorectal cancer (CACC) is crucial to limit its worse prognosis and higher mortality (10). Hence, rising titers of these microRNAs among UC patients may indicate malignant transformation. 

miR-21 and miR-92a can promote cell proliferation, suppress apoptosis of tumor cells, induce tumor angiogenesis, and accelerate its progression in several tumors including CRC, lung, thyroid, breast, and esophageal cancers (18, 36). In addition, these miRNAs serve as oncogenic factors (oncomirs) which can downregulate the proapoptoticBcl-2 and PTEN in CRC (37, 38). Furthermore, miR-21 can downregulate several tumor suppressor genes e.g., PDCD4, MARCKS, and RECK metalloproteinase inhibitor, TPM1 and hence stimulating tumor initiation, invasion, and intravasation. It can also downregulate the core MMR proteins e.g., MSH2, MSH6, and MLH1, inducing the MSI mutator phenotype in CRC (39 – 41). 

One of the limitations of this polite study was its small sample size. Selection of healthy individuals as controls was not blindly assigned, the way practiced in many previous studies. We did not include patients with Crohn's disease or other types of colitis such as diverticulitis because of their scarcity in our locality. In addition, tissue expression levels of miRNAs were not estimated as circulating miRNAs appear to mirror tissue changes. Nevertheless, previous studies found that circulating miRNAs had a promising influence as biomarkers differentiating between functional and organic bowel diseases. Furthermore, a large-scale validation study is required to confirm our findings, to emphasize the mechanism of these biomarkers to either activate or deactivate inflammatory progression pathways and further to clarify their therapeutic role as targets to block disease progression and improve the sensitivity of conventional therapies.

In conclusion, our study highlighted that circulating miR-21 and miR-92a can be exploited not only as potential noninvasive biomarkers for detection of CRC but also for the possibility for differentiation between functional (IBS) and organic colorectal diseases. They can also be used for identification of clinically meaningful colorectal lesions; UC; critical target lesions for CRC screening strategy. Understanding the roles of these miRNAs is important to be incorporated into routine clinical practice in the not-so-distant (or near) future especially for high-risk individuals and identification of new therapeutic targets.

## References

[1] Zhen Y, Luo C, Zhang H (2018). Early detection of ulcerative colitis-associated colorectal cancer. Gastroenterol Rep.

[2] Xavier RJ, Podolsky DK (2007). Unraveling the pathogenesis of inﬂammatory bowel disease. Nature.

[3] Maloy KJ, Powrie F (2011). Intestinal homeostasis and its breakdown in inﬂammatory bowel disease. Nature.

[4] Eaden JA, Abrams KR, Mayberry JF (2001). The risk of colorectal cancer in ulcerative colitis: a meta-analysis. Gut.

[5] Breynaert C, Vermeire S, Rutgeerts P, Van Assche G (2008). Dysplasia and colorectal cancer in inflammatory bowel disease: a result of inflammation or an intrinsic risk?. Acta Gastroenterol Belg.

[6] Kavanagh DO, Carter MC, Keegan D, Doherty G, Smith MJ, Hyland JM (2014). Management of colorectal cancer in patients with inflammatory bowel disease. Tech Coloproctol.

[7] Jess T, Rungoe C, Peyrin-Biroulet L (2012). Risk of colorectal cancer in patients with ulcerative colitis: a meta-analysis of population-based cohort studies. Clin Gastroenterol Hepatol.

[8] Jess T, Simonsen J, Jorgensen KT, Pedersen BV, Nielsen NM, Frisch M (2012). Decreasing risk of colorectal cancer in patients with inflammatory bowel disease over 30 years. Gastroenterology.

[9] Beaugerie L, Svrcek M, Seksik P, Bouvier AM, Simon T, Allez M (2013). Risk of colorectal high grade dysplasia and cancer in a prospective observational cohort of patients with inflammatory bowel disease. Gastroenterology.

[10] Dugum M, Lin J, Lopez R, Estfan B, Manilich E, Stocchi L (2017). Recurrence and survival rates of inflammatory bowel disease-associated colorectal cancer following postoperative chemotherapy: a comparative study. Gastroenterol Rep.

[11] Wang J, Zhang KY, Liu SM, Sen S (2014). Tumor-associated circulating micrornas as biomarkers of cancer. Molecules.

[12] Lewis BP, Burge CB, Bartel DP (2005). Conserved seed pairing, often flanked by adenosines, indicates that thousands of human genes are microRNA targets. Cell.

[13] Iborra M, Bernuzzi F, Invernizzi P, Danese S (2012). MicroRNAs in autoimmunity and inflammatory bowel disease: crucial regulators in immune response. Autoimmun Rev.

[14] Wu WK, Law PT, Lee CW, Cho CH, Fan D, Wu K (2011). MicroRNA in colorectal cancer: from benchtop to bedside. Carcinogenesis.

[15] Khor B, Gardet A, Xavier RJ (2011). Genetics and pathogenesis of inflammatory bowel disease. Nature.

[16] Wu F, Guo NJ, Tian H, Marohn M, Gearhart S, Bayless TM (2011). Peripheral blood microRNAs distinguish active ulcerative colitis and Crohn’s disease. Inflamm Bowel Dis.

[17] Paraskevi A, Theodoropoulos G, Papaconstantinou I, Mantzaris G, Nikiteas N, Gazouli M (2012). Circulating microRNA in inflammatory bowel disease. J Crohns Colitis.

[18] Ng EK, Chong WW, Jin H, Lam EK, Shin VY, Yu J (2009). Differential expression of microRNAs in plasma of patients with colorectal cancer: a potential marker for colorectal cancer screening. Gut.

[19] Tsujiura M, Ichikawa D, Komatsu S, Shiozaki A, Takeshita H, Kosuga T (2010). Circulating microRNAs in plasma of patients with gastric cancers. Br J Cancer.

[20] Kaiser T, Langhorst J, Wittkowski H, Becker K, Friedrich AW, Rueffer A (2007). Faecal S100A12 as a non-invasive marker distinguishing inflammatory bowel disease from irritable bowel syndrome. Gut.

[21] Takagi T, Naito Y, Mizushima K, Hirata I, Yagi N, Tomatsuri N (2010). Increased expression of microRNA in the inﬂamed colonic mucosa of patients with active ulcerative colitis. J Gastroenterol Hepatol.

[22] Yang Y, Ma Y, Shi C, Chen H, Zhang H, Chen N (2013). Overexpression of miR-21 in patients with ulcerative colitis impairs intestinal epithelial barrier function through targeting the Rho GTPase RhoB. Biochem Biophys Res Commun.

[23] Ordas I, Eckmann L, Talamini M, Baumgart DC, Sandborn WJ (2012). Ulcerative colitis. Lancet.

[24] Schroeder KW, Tremaine WJ, Ilstrup DM (1987). Coated oral 5-aminosalicylic acid therapy for mildly to moderately active ulcerative colitis A randomized study. N Engl J Med.

[25] Drossman DA, Hasler WL (2016). Rome IV-functional GI disorders: disorders of gut-brain interaction. Gastroenterology.

[26] Sobin L, Wittekind C (2002). TNM classification of malignant tumors.

[27] Jiang S, Li C, Olive V, Lykken E, Feng F, Sevilla J (2011). Molecular dissection of the miR-17-92 cluster’s critical dual roles in promoting Th1 responses and preventing inducible Treg differentiation. Blood.

[28] Wu F, Dong F, Arendovich N, Zhang J, Huang Y, Kwon JH (2014). Divergent influence of microRNA-21 deletion on murine colitis phenotypes. Inflamm Bowel Dis.

[29] Yang G, Yang L, Wang W, Wang J, Wang J, Xu Z (2015). Discovery and validation of extracellular/circulating microRNAs during idiopathic pulmonary fibrosis disease progression. Gene.

[30] Ludwig K, Fassan M, Mescoli C, Pizzi M, Balistreri M, Albertoni L (2013). PDCD4/miR-21 dysregulation in inﬂammatory bowel disease-associated carcinogenesis. Virchows Arch.

[31] RC (2007). Role of infection in irritable bowel syndrome. J Gastroenterol.

[32] Zhou Q, Zhang B, Verne GN (2009). Intestinal membrane permeability and hyper-sensitivity in the irritable bowel syndrome. Pain.

[33] Zhou Q, Souba WW, Croce C, Verne GN (2010). MicroRNA-29a regulates intestinal membrane permeability in patients with irritable bowel syndrome. Gut.

[34] Kapeller J, Houghton LA, Mönnikes H, Walstab J, Möller D, Bönisch H (2008). First evidence for an association of a functional variant in the microRNA-510 target site of the serotonin receptor-type 3E gene with diarrhea predominant irritable bowel syndrome. Hum Mol Genet.

[35] Ahmed FE, Jeffries CD, Vos PW, Flake G, Nuovo GJ, Sinar DR (2009). Diagnostic microRNA markers for screening sporadic human colon cancer and active ulcerative colitis in stool and tissue. Cancer Genomics Proteomics.

[36] Wang B, Zhang Q (2012). The expression and clinical significance of circulating microRNA-21 in serum of five solid tumors. J Cancer Res Clin Oncol.

[37] Tsuchida A, Ohno S, Wu W, Borjigin N, Fujita K, Aoki T (2011). miR-92 is a key oncogenic component of the miR-17-92 cluster in colon cancer. Cancer Sci.

[38] Humphreys KJ, Cobiac L, Le Leu RK, Van der Hoek MB, Michael MZ (2013). Histone deacetylase inhibition in colorectal cancer cells reveals competing roles for members of the oncogenic miR-17-92 cluster. Mol Carcinog.

[39] Bickeboller M, Tagscherer KE, Kloor M, Jansen L, Chang Claude J, Brenner H (2015). Functional characterization of the tumor-suppressor MARCKS in colorectal cancer and its association with survival. Oncogene.

[40] Sveen A, Agesen TH, Nesbakken A, Rognum TO, Lothe RA, Skotheim RI (2011). Transcriptome instability in colorectal cancer identified by exon microarray analyses: Associations with splicing factor expression levels and patient survival. Genome Med.

[41] Balaguer F, Moreira L, Lozano JJ, Link A, Ramirez G, Shen Y (2011). Colorectal cancers with microsatellite instability display unique miRNA profiles. Clin Cancer Res.

